# Applying Implementation Science to Identify Primary Care Providers’ Enablers and Barriers to Using Survivorship Care Plans

**DOI:** 10.3390/curroncol31060249

**Published:** 2024-06-05

**Authors:** Brittany Mutsaers, Tori Langmuir, Carrie MacDonald-Liska, Justin Presseau, Gail Larocque, Cheryl Harris, Marie-Hélène Chomienne, Lauriane Giguère, Paola Michelle Garcia Mairena, Dina Babiker, Kednapa Thavorn, Sophie Lebel

**Affiliations:** 1 School of Psychology, University of Ottawa, Ottawa, ON K1N 6N5, Canada; jpressea@uottawa.ca (J.P.); lgigu023@uottawa.ca (L.G.); pgarc090@uottawa.ca (P.M.G.M.); dbabi094@uottawa.ca (D.B.); slebel@uottawa.ca (S.L.); 2 The Ottawa Hospital Research Institute, Ottawa, ON K1H 8L6, Canada; tori.langmuir@mail.concordia.ca (T.L.); charris@toh.ca (C.H.); 3 Department of Education, St. Francis Xavier University, Antigonish, NS B2G 2W5, Canada; x2019equ@stfx.ca; 4 Clinical Epidemiology Program, School of Epidemiology and Public Health, University of Ottawa, Ottawa, ON K1N 6N5, Canada; kthavorn@uottawa.ca; 5 Wellness Beyond Cancer Program, The Ottawa Hospital, Ottawa, ON K1H 7W9, Canada; galarocque@toh.ca; 6 C.T. Lamont Primary Health Care Research Centre, University of Ottawa, Ottawa, ON K1N 6N5, Canada; mh.chomienne@uottawa.ca

**Keywords:** survivorship care plans, oncology, primary care, primary care providers, theoretical domains framework, cancer survivorship

## Abstract

Primary care providers (PCPs) have been given the responsibility of managing the follow-up care of low-risk cancer survivors after they are discharged from the oncology center. Survivorship Care Plans (SCPs) were developed to facilitate this transition, but research indicates inconsistencies in how they are implemented. A detailed examination of enablers and barriers that influence their use by PCPs is needed to understand how to improve SCPs and ultimately facilitate cancer survivors’ transition to primary care. An interview guide was developed based on the second version of the Theoretical Domains Framework (TDF-2). PCPs participated in semi-structured interviews. Qualitative content analysis was used to develop a codebook to code text into each of the 14 TDF-2 domains. Thematic analysis was also used to generate themes and subthemes. Thirteen PCPs completed the interview and identified the following barriers to SCP use: unfamiliarity with the side effects of cancer treatment (Knowledge), lack of clarity on the roles of different healthcare professionals (Social Professional Role and Identity), follow-up tasks being outside of scope of practice (Social Professional Role and Identity), increased workload, lack of options for psychosocial support for survivors, managing different electronic medical records systems, logistical issues with liaising with oncology (Environmental Context and Resources), and patient factors (Social Influences). PCPs value the information provided in SCPs and found the follow-up guidance provided to be most helpful. However, SCP use could be improved through streamlining methods of communication and collaboration between oncology centres and community-based primary care settings.

## 1. Introduction

Cancer survivors have specific and changing needs as they complete treatment and move into follow-up care [1,2,3,4]. These include regular surveillance for new or recurring cancers, management of the side effects of treatment (e.g., neuropathy, fatigue), providing emotional support, and managing other health issues. The breadth and individualized nature of follow-up cancer care has led to new models of cancer care being developed to meet the needs of cancer survivors [5,6,7]. For certain survivors with fewer needs requiring specialists’ care, primary care providers (PCPs) may be an appropriate health care provider to manage follow-up care [6]. The rationale for this includes the following: the nature of follow-up care is in the purview of PCP tasks, PCPs have long standing relationships with their patients, and PCP follow-up is a way to allocate healthcare resources so that oncology centres can focus on acute treatment and new diagnoses [1,2,8,9,10]. 

However, the transition back to primary care post-cancer treatment can be challenging. Survivors tend to feel a mix of relief that they are being discharged but also a fear of losing the support of oncology specialists [1,11]. PCPs play a key role in facilitating this transition. They are often the health care professionals who found the cancer and have expertise in managing chronic illness [7]. That said, cancer survivors have specific follow-up cancer care needs that are now the responsibility of PCPs. Additional supports are needed to equip PCPs with the information, resources, and connection to oncology specialists needed for a successful post-treatment transition [1]. Survivorship programs and survivorship care plans (SCPs) can bridge the gap between tertiary and primary care [2].

The Wellness Beyond Cancer Program (WBCP) is one such survivorship program associated with the Ottawa Hospital Cancer Centre in Ottawa, Ontario, Canada [2]. This nurse-led program is geared toward breast and colorectal cancer survivors deemed low-risk and discharged by their oncologists back to primary care for follow-up. One of the resources the WBCP provides is individualized SCPs that are provided to both cancer survivors and their PCPs. SCPs outline key information about the individual’s cancer history and treatments, the identification of specific needs (e.g., chronic fatigue, psychological distress), healthy lifestyle behaviours recommended, and instructions for follow-up care. Instructions generally include the frequency of surveillance tests (mammograms, colonoscopy), recommendations for managing late- and long-term cancer treatment side effects, signs and symptoms of recurrence, and contact information of local oncology centres [2].

Although SCPs are recommended by several cancer organizations [8,12,13], there is a lack of consistent evidence to support their use [14,15]. Two systematic reviews of research studies of the impact of SCPs have outlined large differences in the content, format, and delivery of SCPs, inconsistent or irrelevant outcomes used to measure their effectiveness, and the possibility that SCPs are not useful for PCPs and cancer survivors for facilitating the transition back to follow-up care [14,15]. 

This is in contrast with previous studies of PCPs’ perceptions of SCPs that have found that they are accepting of SCPs as transition tools [16] because they contain the information needed to provide follow-up cancer care, which increases their comfort in taking on the role [17]. That said, potential barriers to SCP use among PCPs included issues around funding for providing follow-up care, concerns about their level of knowledge and confidence in providing follow-up care for cancer survivors, lack of time, issues with using different electronic medical records, and an already high workload [18,19]. On the other hand, PCPs have described factors increasing the usability of SCPs, such as a length of one page, clear and easy to find guideline recommendations, and developing systems for setting up follow-up care reminders [20].

### Aim of the Study

Before abandoning SCPs, more understanding is needed in how they are being implemented in routine practice. An important step is to investigate how SCPs have been used by PCPs, one of the end-users of SCPs, to find ways to improve their uptake and usability as transition tools. The purpose of this study is to systematically identify enablers and barriers of SCP use among PCPs using implementation science. This study is part of a larger project that aimed to determine best practices around SCPs from the perspectives of multiple stakeholders [21]. 

## 2. Materials and Methods

### 2.1. Choosing an Implementation Science Framework

As reviewed by Nilsen (2015), there are several implementation science theoretical models and frameworks to consider. For this study, we chose a determinant framework, as these frameworks were developed to help identify how different barriers and enablers impact behaviours and related outcomes. These frameworks also allow for the consideration of how barriers and enablers interact and can be modified to achieve a different or preferred outcome [22]. The Theoretical Domains Framework (TDF) is a determinant framework initially with 12 different domains that help systematically look for barriers and enablers to desired behaviours [23]. The TDF was validated, and a revised version (TDF-2) was published in 2012 where 10 domains from the initial version of the TDF were unchanged, the domain “Nature of the Behaviours” was removed, and the domains Optimism, Reinforcement, Goals, and Intention were added [24]. The TDF has been used to study desired behaviour in implementation projects related to changing healthcare practice, lifestyle change programs, and mental health supports [25]. A manual has been developed and outlines how to define the behaviours being examined, collect and analyse data, and report the results [25]. The TDF-2 was chosen for this study because it is a standardised way of systematically identifying barriers and enablers to SCP use among PCPs.

### 2.2. Materials

A semi-structured interview guide was developed based on TDF-2 [24]. Its 14 different domains allow for the systematic assessment of the enablers and barriers to specific behaviours [25], in this case, PCPs using SCPs. First, a minimum of one question per domain was created and revised by the research team to include open-ended questions with prompts to avoid a sense of repetitiveness [25]. Questions for PCPs included “Did you feel you had the necessary information to provide follow-up care for your patient?” (TDF domain: Knowledge) and “What would make it easier to use SCPs?” (TDF domain: Behavioural Regulation). See Supplementary Materials for the interview guide. 

### 2.3. Participants 

For PCPs, the inclusion criteria were (a) a general practitioner/family medicine physician or a nurse practitioner; (b) had received at least one SCP from the WBCP at least 12 months prior to the interview (to ensure they had time to have used the SCP); (c) could speak and understand English or French; and (d) provided consent to participate in a one-time 15–20 min telephone interview. 

### 2.4. Recruitment and Procedure 

PCPs were recruited from a database of PCPs who were sent at least one SCP because they were identified as providing care to breast or colorectal WBCP patients. They were mailed invitations to participate in an interview. Because PCPs are highly difficult to recruit [26], PCPs were also recruited by networking among PCPs, including telephone or email invitations by known PCPs (i.e., co-investigator MHC). PCPs who were interested in participating contacted the research team through telephone or email communications, and the consent form was reviewed and verbal consent was obtained over the telephone. A copy of the consent form was mailed or emailed to participants based on their preference, and a copy of the interview questions was provided ahead of the interview if the participant wanted to review them before the interview. Interviews were conducted over the telephone as per public health restrictions during the COVID-19 pandemic, and then they were audio-recorded and transcribed. PCPs were compensated with a $100.00 gift card, which was mailed to them after the interview. 

### 2.5. Data Analysis

To promote the consistency of the text coded into each of the 14 TDF domains, BM and TL created a codebook to guide initial content analysis. Within each of the TDF domains, text with similar concepts were grouped together and represented with a “Belief Statement” [25,27]. According to Atkins et al. [25], “a belief statement is a collection of responses with a similar underlying belief that suggest a problem and/or influence of the beliefs on the target implementation problem”, in this case, PCPs’ use of SCPs in providing follow-up care to cancer survivors. As recommended by Atkins et al., each belief statement was counted once within each interview to generate a frequency count across all interviews within each TDF domain. Lastly, using thematic analysis, themes and subthemes were identified across the TDF domains [28]. The 10+3 rule for data saturation [29] guided the recruitment efforts. This “rule” has been used as a way of considering data saturation in qualitative methods used in implementation research [29]. This rule has been recommended for use with the TDF [25], and it suggests that after 10 interviews, additional interviews should occur in groups of three until no new information is found in these subsequent interviews [29]. To increase the trustworthiness of the data analysis, four PCP interviews were independently double-coded into the 14 TDF domains by BM and TL. The Krippendorf’s Alpha statistic was used to measure inter-rater reliability and was 0.89, which indicates that there was high agreement and reliability in the coding [30]. 

### 2.6. Ethical Approval

Ethics approval was provided by the Ottawa Health Science Network Research Ethics Board (file # 20200152-01H). 

## 3. Results

Thirteen PCPs completed an interview; all were medical doctors. The average number of years in practice reported at the interview was 15 years (range 5 to 38 years). The average duration of the interviews was 21 min (range 14 to 30 min). After 10 interviews, an additional 3 were conducted and no new information arose, suggesting that data saturation was reached [29]. Knowledge, Social Professional Role and Identity, Environmental Context and Resources, Social Influences, and Behavioural Regulation were the most prevalent TDF domains for the use of SCPs by PCPs. Table A1 and Table A2 outline the enablers and barriers of SCP use, respectively, along with their accompanying belief statement. 

Three themes emerged from the data: (1) SCPs are helpful to arrange follow-up care, but there are logistical barriers that impede their usefulness; (2) SCPs can facilitate collaboration between PCPs and survivors; and (3) SCPs can help PCPs accept the role of providing follow-up care. Table A3 outlines the TDF domains within each theme, with Knowledge, Social Professional Role and Identity, Environmental Context and Resources, and Emotion being the most frequent domains.

**Theme** **1.** **SCPs are helpful to arrange follow-up care, but there are logistical barriers that impede their usefulness.**

**Subtheme 1: SCPs contain valuable information from a credible source.**

PCPs stated that the information included in the SCP is highly relevant and useful to coordinate follow-up cancer care for their patients. They found the follow-up care instructions summarized in a table at the end of the SCP particularly useful:

*“The other information is good if I need to do a deep dive, but 90 percent of the time I'm looking at the note, I don't need to do a deep dive. I need to, in the middle of a meeting, go back and say, “OK, this was related to your cancer. Let's go back and just see what the cancer care guide said about how we're supposed to pull this out. OK, great. This is our follow-up, boom, and we're going to do this.” If I have to wade through 10 pages to figure out what my actions were, that's not a useful note to me”*.

(PCP 13)

They also found the follow-up information reassuring because they knew it was provided by oncology specialists:

*“…as family doctors, we're not always able to be, you know, right up to date with the most recent guidelines and recommendations for, you know, each type of cancer, the appropriate follow up, so, having that road map that's provided in the document is key, just to make sure that I know that I'm providing the care that, you know, the level of care that I'm hoping to provide to my patients and that they're being followed appropriately”*.

(PCP 8)

PCPs used SCPs as a starting point to discuss the process of follow-up care with their patients and as a framework for organizing themselves to provide appropriate follow-up care. For example, the receipt of an SCP prompted PCPs to set reminders for appointments based on the recommended guidelines. PCPs noted that performing the tasks outlined at the end of the SCP was most of the time relatively easy. 

**Subtheme 2: There are logistical barriers that decrease the usefulness of SCPs.**

While PCPs found the recommendations of the SCPs useful, many mentioned that ordering follow-up tests was a reportedly convoluted process:

*“…with regards to guidelines being easy, from my perspective, that once it goes on to colonoscopy, it's no longer easy and guidelines and recommendations are very conflicting”*

(PCP 10)

They also described how they had concerns about guidelines changing in the future, something that is not covered with the current SCP format:

*“I’m also aware that the guidelines will change, right? So, what is, for the Wellness Beyond Cancer Program plan that you sent me now, you know, I’m still ordering things in five or ten years, but then, you know, will that still be up-to-date, right? I don’t have access and I wouldn’t know where to find the guidelines or how to implement them”*

(PCP 6)

Additionally, PCPs identified aspects of providing follow-up care that require more clarity in the SCPs. For example, when follow-up care tasks should end:

*“So, you know, really, what do I need to know, you know, keep it to a minimum what I need to know, but more importantly, what do I need going forward? So, if it’s a certain exam, if it’s a mammogram, it’s knowing how long they need to be on their hormone replacement therapy”*.

(PCP 11)

Last, PCPs described the benefits and challenges of using the electronic medical record (EMR) in providing follow-up cancer care. They stated that they used their EMR to set reminders for when follow-up tests need to be ordered, which was useful. However, the process of getting the SCP integrated into the PCPs’ specific EMR can be a time-consuming barrier to optimal use:

*“And it’s impractical that the care plan is not integrated in the EMR, but it’s fine, you know, I would still have to set myself a bunch of reminders, so I take 15-20 minutes, go through the care plan, set all my reminders, then I’m good to go, right?”*

(PCP 6)

**Subtheme 3: PCPs need connection with oncology specialists to fully benefit from SCPs**

PCPs noted that while the information in the SCP contains helpful guidance, they were unsure of the process around consulting cancer specialists, especially regarding unusual test results:

*“…having that be clearer on the wellness paper that we get that, you know, “here's how you would go about, you know, calling us and contacting us if you have any concerns or questions about, you know, the patient's ongoing care needs”*.

(PCP 8)

It appeared that, in some instances, the contact information that PCPs can use to consult with oncology specialists is included in discharge notes, rather than in the SCP specifically, which was an additional burden on the PCP.

*“Yes, exactly, whenever I receive anything, and I think the oncologists write it at the end of their notes, like, “if you have any questions or if anything changes, you are more than welcome to contact us at this number for this information”, so I always keep it with me, making sure that in case I need it, I can contact”*.

(PCP 5)

However, once communication was established with oncology specialists, either by PCPs and/or by cancer survivors, it often became circular. This was described by one PCP:

*“Yeah, and I also find the notes, and I think it's in the cancer plan, but the note in the discharge plan says “for any questions, just contact your primary care provider”, and sometimes we’re not very well-equipped to answer these questions, right, like they’ll ask me a question and I’m like, “I don’t know, my answer would be to call them back”, because they’re the cancer specialists and this is all they do, but then they run into the problem of “well, when I call them, they said to call you” and I’m like, yeah okay, I’m not gonna throw you back to them, I guess I can deal with it myself”*.

(PCP 2)

To address their information needs, PCPs reported they frequently used an e-consult service when they had questions for specialists, highlighting their need for prompt communication with oncology specialists:

*“…when the primary care provider has a question specifically for a specialist, and we will – we can write online, the only issue with E-consult is it’s great but it’s not in our EMR. If I have a question for example, I have this patient, cancer survivor, has had this type of breast reconstruction surgery, what type of mammogram should I be ordering? Like what type of frequency, what kind of sites are able to do the mammogram, and things like that, so I’ll send it off to oncology, and sometimes there’s like, oncology breast, or oncology lung, so you will submit these types of question to a specialist and they will respond within a week or two weeks, which is really great, we get a rapid response”*.

(PCP 6)

A similar challenge with access to appropriate providers and resources was mentioned by participants when they described how they attempted to manage the psychosocial concerns raised on the SCP:

*“So, that's a burden that unfortunately all family docs have to overcome with all of their patients in a world where there's very little to offer for free, for mental health, so unfortunately that’s all of us that struggle with that. And definitely there's no obvious solution any time soon”*.

(PCP 10)

Overall, PCPs described finding SCPs a helpful source of information and guidance but face logistical barriers that can be improved upon to make providing follow-up care a smoother process, especially around accessing oncology specialists when needed.

**Theme** **2.** **SCPs can facilitate a give and take relationship with regard to follow-up care** **.**

PCPs described how they collaborated with their patients to whom they are providing follow-up care. For example, they described how having an SCP helped them take on the responsibility of ensuring the patient adheres to the follow-up schedules:

*“it puts a lot more of the ownership on us to make sure that we’ve ordered the tests and received the tests, because a lot of the time patients will assume that no news is good news, so it kind of just forces me to do a lot more sort of background checking, and you know, setting reminders in my EMR to look out for those results in 6 months' time, was it ordered, was it done? So, it does force us to be more proactive in ordering, especially for checking”*.

(PCP 7)

PCPs empathized with the emotional experiences of cancer survivors, including how some survivors may feel hesitant about leaving the cancer centre and how SCPs can help with this. For example, one PCP said the following:

*“I think if the patient is just discharged and saying, you know ‘Go see your family doctor if anything comes up’ and, you know, it would be - that would be that would be very, I think that would cause a lot of anxiety on the physician's part and the patient's part. I think the care plan is really helpful”*.

(PCP 9)

PCPs highlighted how some of their patients use their SCPs to initiate appointments:

*“…usually people are pretty good at remembering that, so, there’s a back-up because I have it in my file, and the patient has it in hers, you know, so it’s unlikely that we’re going to miss, so I don’t think scheduling is a big problem, because there’s two of us that put in reminders in our agendas”*.

(PCP 4)

Indeed, the importance of patients taking some responsibility for their follow-up care was echoed by several PCPs. For example, one PCP said the following:

*“I also try – like, most of my patients are quite good, they’re also young, so mostly I also leave it a lot up to them, to say like, “look, I have a message in the chart, but it’s also your responsibility to at least check in with me every 6 months, so that when I see your name and I go into the chart, then I have that reminder to be like, oh, okay, it’s November, we’re due to do like, all of this stuff”*.

(PCP 2)

To summarize, PCPs see the SCP as an important tool for both themselves and cancer survivors and expect that survivors will take on some aspects of their follow-up care.

**Theme** **3.** **SCPs can help PCPs accept the role of providing follow-up care**

PCPs accepted the role of providing follow-up cancer care, said that it made sense because of their ongoing relationship with their patients, and felt mostly positive about the role. However, there was an acknowledgment of increased workload:

*“I mean, there’s probably some resentment in that, you know, it’s now, all of a sudden there’s more work, for what I was considering at that point a shared patient, but you know, in a system like ours, I recognize that there is going to be a certain amount of downloading so that we can preserve resources for the most appropriate use, and if the patient doesn’t have cancer, there isn’t really any reason for them to see cancer doctors”*.

(PCP 3)

It appeared that for some PCPs, SCPs facilitated the role of providing follow-up care by decreasing uncertainty:

*“Yeah, so, being a family doctor that whenever we receive the cancer survivors and all the details and you know, their – how to care for them, like in the future, I feel like it strengthens my relationship with the patients because they feel like they’re not just left alone, because once they stopped with their oncologist, like their wellness care, they have this ongoing support from me. And that’s not only important for them, but for myself too, right, because I know what exactly is happening and how I would be helping them in the future”*.

(PCP 5)

*“I mean, that's basically been my experience is that, you know, being a primary care physician, you often are not sure, depending on the type of cancer, what sort of frequent, what sort of follow up is expected and what frequency is expected and so on. And that plan is usually helpful in relieving a lot of the uncertainty from the physician point of view, but also the insecurity from the patient point of view”*.

(PCP 9)

Overall, SCPs appeared to be a helpful tool to facilitate PCPs’ role in providing follow-up care. 

## 4. Discussion

The present study focused on systematically identifying enablers and barriers to SCP use by PCPs using the TDF-2, a validated implementation science framework. Barriers were primarily related to additional workload due to a lack of clarity around the best procedures for ordering and receiving follow-up test results. Contact with oncology specialists was needed throughout the process of providing follow-up care to optimise the use of SCPs. Although PCPs did acknowledge the increased workload associated with being assigned the role of providing follow-up care, any negative experiences or emotions reported were related to environmental barriers within the healthcare system (e.g., importing the SCP into the EMR). The SCP itself was described as helpful and included relevant information in an easy-to-follow format. PCPs found that it was a helpful tool to discuss follow-up care with their patients and that patients also having a copy of the plan facilitated the timely scheduling of follow-up appointments. Moreover, patients being prepared to share the responsibility of follow-up care appeared to be a facilitator of them bringing their SCPs and being proactive about their follow-up tests. Overall, the SCP was an important source of information about follow-up care, but additional supports are needed to increase their usefulness. 

### 4.1. Allocation of Health Care Resources: The Role of PCPs

In risk-stratified models of care, PCPs have been deemed appropriate to support low-risk cancer survivors who can self-manage their care after active treatment [6,7]. Survivors with more complex needs are also discharged back to primary care, where their care could be shared between PCPs and oncology specialists [5,6,7,31]. To implement these models of care, care planning, information provision, and ways to coordinate care are needed [31]. Systematic reviews have found that the following factors are important in facilitating good health outcomes: (a) clear avenues of communication; (b) planning ways to meet the needs of survivors; and (c) ensuring guidelines for each cancer type are followed [31]. The PCPs interviewed in the present study found SCPs helpful in guiding follow-up cancer care, but additional supports are needed to address barriers to their use. As reflected in the literature, PCPs need more education and information on cancer-specific issues, despite having experience managing other chronic illnesses [6,31]. Many PCPs described the need to consult with oncology specialists on an ongoing basis. In line with these results, authors have argued that it is not realistic to expect PCPs to be able to manage follow-up care without the input of specialists. Despite wanting to learn more about cancer survivorship, other demands on PCP’s time can interfere with their ability to do so [5,31]. 

When considering ways to promote the use of SCPs, the results indicate that factors within the health care system itself must be examined. As highlighted in a systematic review of new models of care for cancer survivorship, a key facilitator of implementation was offering a range of options for communicating and coordinating care [31]. More specifically, application-based, virtual/telehealth, and other technologies can streamline the process of communication among PCPs, oncologists, and patients [6,31]. Preliminary work on videoconferencing between PCPs and oncologists suggests that such methods of communication can facilitate the provision of follow-up care in a timely fashion that specifically meets the individual needs of survivors [32]. Furthermore, consistent policies around how best to order follow-up screening for new and recurrent cancers and consulting oncology specialists when survivorship needs are out of scope for PCPs to manage are needed [6,33]. 

### 4.2. Future Research

While PCPs’ role in different models of follow-up care have continued to be explored [6], SCPs appear to be a valuable source of information for PCPs. Additional research is needed on ways to improve their integration into the daily practice of PCPs such that the addition to their workload is minimized. Streamlined approaches to communication between PCPs and oncology specialists is needed to facilitate PCPs’ ability to follow through with test ordering, the receipt of results, and the steps that need to be taken should test results indicate that further action is required. 

To address these specific barriers and promote the use of SCPs among PCPs, researchers could generate implementation strategies to change behaviours associated with SCP use. For this, future studies could use another implementation framework, the Behaviour Change Technique Taxonomy [34,35,36], which is made up of 93 specific and precise definitions of strategies to change behaviour [34,35] and is one of the most researched and expert-approved taxonomies for developing implementation interventions [36]. A tool exists to facilitate the link of each TDF domain to specific strategies from the BCTTv1 [36].

### 4.3. Strengths and Limitations

The barriers and enablers of SCP use were identified based on the perspectives of PCPs, who are one of the intended end-users of SCPs [2]. Participants were familiar with SCPs and had used them prior to the interview, which is an important strength. Data saturation was reached based on the guidance from the 10+3 rule [29]. The research team developed a detailed codebook outlining the decision rules for organizing texts into each of the 14 TDF domains, which facilitated a high inter-rater reliability, suggesting that the analysis was consistent across independent coders [25,30]. Limitations included the small sample size and the inclusion of PCPs from the Ottawa area only, which may limit the generalizability of the barriers and enablers identified. 

## 5. Conclusions

SCPs provide succinct information that facilitates the provision of follow-up cancer care. Within the overall context of finding ways to ensure cancer survivors receive the follow-up care they need, SCPs have a relevant role in coordinating follow-up care between PCPs, oncology specialists, and cancer survivors but barriers need to be addressed to increase their usefulness. 

## Data Availability

The dataset is available on request from the authors.

## Supplementary Materials

The following supporting information can be downloaded at: https://www.mdpi.com/article/10.3390/curroncol31060249/s1, File S1: Interview Guide for Primary Care Providers.

## Appendix A

**Table A1:** Summary of Enablers to SCP Use by PCPs.

TDF Domain	Belief Statement	#PCPs Reporting
Knowledge	SCP has follow-up care guidelines	7
	Information in SCP is relevant	10
	SCP outlines tasks for PCPs	10
Social Professional Role and Identity	I book tests, appointments, respond to concerns, etc.	13
	I can solve problems using the SCP	2
	Patients should take responsibility too	7
	Oncology specialists create the plan, so I follow it	4
	I’m used to providing follow-up cancer care	9
Beliefs About Capabilities	Providing follow-up cancer care is easy	8
	I can handle the responsibility of providing follow-up cancer care	3
Beliefs about Consequences	Using SCPs will lead to a positive outcome	2
Memory, Attention and Decision Processes	I use the SCPs to create reminders for myself	9
Environmental Context and Resources	Layout of SCP makes things clear	5
Social Influences	I keep the patient on schedule	5
	I provide patients with the support they are asking for	6
	Patients remind me when follow-up tests are needed	4
Emotion	PCP’s positive emotions about providing follow-up care	5

**Table A2:** Summary of Barriers to SCP Use by PCPs.

TDF Domain	Belief Statement	#PCPs Reporting
Knowledge	I don’t know what treatments my patient is receiving	7
	Not knowing about side effects of cancer-specific medication	5
Social Professional Role and Identity	I need to defer to specialists	9
	I don’t know what to do if test results are abnormal	2
	I don’t know who is responsible for a task	4
	Some tasks of follow-up cancer care are outside of my scope of practice	3
Beliefs About Capabilities	Challenges providing follow-up care	7
Beliefs about Consequences	Providing follow-up care adds to my workload	4
Environmental Context and Resources	Challenges with finding psychosocial support for patients	6
	I don’t have the SCP	2
	Logistical issues	11
	Using e-consult	3
Social Influences	Patient emotions affects SCP use	7
	Patient intentions affect SCP use	8
	Patients miss follow-up appointments	3
Emotion	Impressions of patients’ feelings about receiving follow-up cancer care from PCP	10
	PCP’s negative emotions around providing follow-up care	5
Behavioural Regulation	Clarity needed on expectations for follow-up cancer care	3
	Suggestions to improve format or delivery of SCPs	7
	More information needed on psychosocial supports available to patients	5
	Descriptions of the type of information that would be helpful to include in SCPs	6
	Patient education on follow-up cancer care is needed	3
	PCPs wanting a streamlined way to contact oncology specialists	2
	Develop ways to update plan or patient information	5

**Table A3:** TDF Domains Within Each Theme.

Theme	TDF Domains
(1) SCPS are helpful to arrange follow-up care but there are logistical barriers that impede their usefulness	Knowledge Social Professional Role and Identity Beliefs about Capabilities Environmental Context and Resources Behavioural Regulation
(2) SCPs can facilitate collaboration between PCP and survivor	Social Professional Role and Identity Environmental Context and Resources Social Influences Emotion
(3) SCPs can help PCPs accept the role of providing follow-up care	Social Professional Role and Identity Beliefs about Consequences Intention Memory, Attention and Decision Process Environmental Context and Resources Emotion
