# Supplementary material for: Applying Implementation Science to Identify Primary Care Providers’ Enablers and Barriers to Using Survivorship Care Plans
Source: Curr Oncol. 2024 Jun 5;31(6):3278–90. doi: 10.3390/curroncol31060249 (PMC11202923; doi:10.3390/curroncol31060249)
Supplement: Supplementary file 1 [file curroncol-31-00249-s001.zip › curroncol-2996002-supplementary.pdf]

<u>Interview Guide for Primary Care Providers</u>

ID:	
Provider Type<br><br># Years in practice:	☐ General Practitioner (MD)<br>☐ Nurse Practitioner (NP)
Location (City/town)	☐ Urban<br>☐ Rural<br>☐ Remote
Received a SCP from Wellness Beyond Cancer Program before?	☐Yes<br><br>☐No

1. Tell me about your experience providing follow-up cancer care for your patients?
   - How do you feel about providing follow-up care to cancer survivors?
   - What are the challenges associated with taking on this responsibility?
     - *Prompt if necessary*…do you feel comfortable? Is this an added task you had not previously encountered? Do you feel this was somewhat imposed?
   - Did you feel you had the necessary information to provide follow-up care for your patient?
   - Did your patient feel comfortable with receiving follow-up care from you? Did he/ she have any concerns?

2. As a primary care provider, how do you manage the follow-up care for your patients who have been discharged from the cancer centre?
   - Prompt: Meeting with cancer survivor patients, conducting history and physicals, ordering surveillance tests; referring to other health care professionals etc.
   - Prompt: Any time, resource, or logistical issues?
   - Prompt: Do you know of any guidelines /do you use any guidelines recommendations for follow-up cancer care? Any tools? What is your source? (Where did you find them)
     - If you do not use any guidelines – would guidelines be useful to you?
     - Do you feel the Cancer Centre should be providing you with those guidelines?
   - Are you comfortable or sufficiently aware of the side effects of their medication
   - How do you approach cancer survivors' psychosocial concerns related to follow-up care (i.e., anxiety, depression, fear of recurrence, sexual concerns, etc.)

3. Have you used patients' survivorship care plan in providing follow-up care?
   - Yes
     - How do you typically use the SCP?
     - How have SCPs been useful? Less useful?
     - Which parts of the SCP did you find most useful?
     - What gets in the way of using a patient's SCP?
       - Prompt: What led to the decision to not use a SCP?

- What would make it easier to use SCPs
    - Prompts: different format; change in content; more time, etc.
- Are there situations where you would probably not use a patient's SCP? Why?

4. Based on your experience, do you have any suggestions or strategies that you would recommend for how we could go about implementing the use of SCPs in primary care?
    - What are the most important factors that would influence you to use SCPs when providing follow-up cancer care to your patients?
        - Prompt: What do you think is needed to ensure that SCPs are consistently used for follow-up cancer care?

Is there anything else you'd like to expand on?
